# Multifactor Analysis of a Genome-Wide Selection System in *Brassica napus* L.

**DOI:** 10.3390/plants14142095

**Published:** 2025-07-08

**Authors:** Wanqing Tan, Zhiyuan Wang, Jia Wang, Sayedehsaba Bilgrami, Liezhao Liu

**Affiliations:** 1 Integrative Science Center of Germplasm Creation in Western China (CHONGQING) Science City, College of Agronomy and Biotechnology, Southwest University, Chongqing 400715, China; 2 Academy of Agricultural Sciences, Southwest University, Chongqing 400715, China

**Keywords:** *Brassica napus*, deleterious mutation, genome-wide selection (GS), prediction accuracy (PA)

## Abstract

*Brassica napus* is one of the most important oil crops. Rapid breeding of excellent genotypes is an important aspect of breeding. As a cutting-edge and reliable technique, genome-wide selection (GS) has been widely used and is influenced by many factors. In this study, ten phenotypic traits of two populations were studied, including oleic acid (C18:1), linoleic acid (C18:2), linolenic acid (C18:3), glucosinolate (GSL), seed oil content (SOC), and seed protein content (SPC), silique length (SL), days to initial flowering (DIF), days to final flowering (DFF), and the flowering period (FP). The effects of different GS models, marker densities, population designs, and the inclusion of nonadditive effects, trait-specific SNPs, and deleterious mutations on GS were evaluated. The results highlight the superior prediction accuracy (PA) under the RF model. Among the ten traits, the PA of glucosinolate was the highest, and that of linolenic acid was the lowest. At the same time, 5000 markers and a population of 400 samples, or a training population three times the size of an applied breeding population, can achieve optimal performance for most traits. The application of nonadditive effects and deleterious mutations had a weak effect on the improvement of traits with high PA but was effective for traits with low PA. The use of trait-specific SNPs can effectively improve the PA, especially when using markers with *p*-values less than 0.1. In addition, we found that the PA of traits was significantly and positively correlated with the number of markers, according to *p*-values less than 0.01. In general, based on the associated population, a GS system suitable for *B. napus* was established in this study, which can provide a reference for the improvement of GS and is conducive to the subsequent application of GS in *B. napus*, especially in the aspects of model selection of GS, the application of markers, and the setting of population sizes.

## 1. Introduction

With the rapid development of genomics, 3517 genomes of 1575 plant species have been sequenced as of 2023, of which the number of genomes sequenced in 2021–2023 reached 2373, and the number of resequenced genomes is countless [1]. One of the most important oilseed crop species in the world, *Brassica napus* (rapeseed), completed its genome sequencing in 2014 [2]. In recent years, many *B. napus* populations have been resequenced and accompanied by the investigation of different phenotypes, with the main objective of combining phenotypic and genotypic data for GWAS to identify candidate genes for target traits [3,4,5,6,7]. Nevertheless, the selection of elite varieties remains an important part of breeding. At the same time, the determination of several traits, such as the unsaturated fatty acid content, is relatively complicated. As a cutting-edge and reliable technique, genome-wide selection (GS) can quickly reveal phenotypic information of the breeding population (BP) based on that of the training population (TP) [8]. Moreover, the genome of one rapeseed germplasm can provide a reference for almost all of its phenotypic information, which would facilitate the selection of integrated superior varieties. Previous studies have applied GS for flowering time, seed quality traits, and blackleg resistance, and confirmed its effectiveness in the *B. napus* breeding program [9,10,11]. Studies in other species have demonstrated that the prediction accuracy (PA) of GS is affected by many factors [12,13,14]. However, a systematic and comprehensive analysis of GS in *B. napus* is still lacking [15,16,17,18].

Statistical models, number of markers, population size, ratio of TP and BP, nonadditive effects, and deleterious mutations have been reported to affect the PA of GS. Currently, there are various GS models available, including genomic best linear unbiased prediction (GBLUP) [19], ridge regression BLUP (RR-BLUP) [20], BayesA, BayesB [21], reproducing kernel Hilbert space (RKHS) [22], and random forests (RF) [23]. These models have been widely used in many plants, the predictive abilities of different methods are inconsistent across species and traits, and no single method can provide a universally optimal fit to all data [10,12]. In fact, higher marker densities ensure that trait-associated QTLs have at least one marker in the LD, resulting in higher prediction performance [24], but higher marker densities are also linked to more computing time and costs. On the other hand, using a small number of significant markers as fixed effects in GS models can achieve more accurate predictions [25,26]. The design of TPs also plays an important role in GS [12,27]. Most classical approaches to GS are dominated by additive effects [28]. Nonadditive effects, including dominant (D) and epistatic (E) effects, can substantially impact phenotypes and are particularly important in polyploid plants [29]. In potato, the inclusion of nonadditive effects in the GS model improved the PA of yield, specific gravity, and potato color [30]. Consideration of nonadditive effects has also been shown to improve PA in wheat and sorghum studies [31,32]. Recently, a study on hybrid potato showed that the inclusion of deleterious mutations improved the ability of GS to predict complex fitness-related agronomic traits controlled by multiple minor effector loci [33].

To obtain a better understanding of the influencing factors of GS and promote the application of GS in *B. napus*, this study used ten phenotypic traits of two associated populations as the basis for statistical analysis of PA with different GS models, numbers of markers, marker types, TP designs, and the inclusion of nonadditive effects and deleterious mutations. The main objectives of this study were to evaluate the ability of GS to predict ten phenotypic traits, evaluate the influence of different factors on the PA, and provide suggestions for subsequent breeding practice of *B. napus*.

## 2. Materials and Methods

### 2.1. Plant Materials and Phenotypic Traits

Two populations were used in this study, including the 60 K population and WGR population, which contained 520 and 604 accessions, respectively [5,34,35,36]. The detailed information about the accessions is in Tables S1 and S2; the 60 K population was collected from China and overseas and contained spring and semi-winter accessions. The WGR population was divided into three ecotypes, and both populations were divided into two subpopulations. Two populations were planted at Southwest University of Beibei (29°45′ N latitude, 106°22′ E longitude, and an altitude of 238.57 m), Chongqing, China. Six seed quality traits, including oleic acid (C18:1), linoleic acid (C18:2), linolenic acid (C18:3), glucosinolate (GSL), seed oil content (SOC), and seed protein content (SPC), were detected by near-infrared reflectance spectroscopy (NIRS) analysis in 2013 and 2014. Silique length (SL) was measured in 2015 and 2017 [36]. Three growth period-related traits were measured in this study, namely, days to initial flowering (DIF), days to final flowering (DFF), and the flowering period (FP). These phenological traits were investigated in 2017, 2018, and 2019, and the BLUE values were used for analysis [37]. The six seed traits corresponded to the 60 K population, and DIF, DFF, FP, and SL corresponded to the WGR population.

### 2.2. Genotypic Data Analysis

According to the criteria of a minor allele frequency (MAF) greater than 0.05, the number of missing markers less than 0.2, and the samples with missing markers less than 0.2, the raw SNP data were filtered through PLINK1.9 [38]. Finally, the 60 K and WGR populations contained 31,725 and 385,853 high-quality markers, respectively.

### 2.3. Genome-Wide Selection Models

In this study, four GS models, namely, BayesB, GBLUP, RKHS, and RF, were selected for comparison. The GBLUP model is described as:

y = Xb + Zu + e

where y is the vector of phenotypic traits, e is the vector of random residuals with variance of Iσ^2^_e_, and I is an identity matrix. b represents the fixed effects, and u represents the random effects with u~N (0, Gσ^2^_u_), where G is the genomic relationship matrix calculated using marker information. X and Z are design matrices for fixed effects and random effects, respectively.

The Bayes models were described as:

y = Xβ + Zu + ε

different Bayes models have different prior distributions. The BayesB model assumes that the effects of most markers (π) are zero, and only a few markers have a significant effect.

These two models, the semiparametric model RKHS and the nonparametric model RF, were implemented by the R package “BWGS” in R 4.3.3 under default parameters [39]. The PA was regarded as the correlation between the genomic-estimated breeding values (GEBVs) obtained by GS and the phenotype values, and the models were evaluated by fivefold cross-validation. Four models were repeated 100 times.

### 2.4. GS Practices with Different Marker Densities

Due to the difference in the time required by different models, the GBLUP model was used to compare the PA between different marker densities. A total of 100, 500, 1000, 5000, and 10,000 markers were randomly selected using the code “geno.reduct.method = “RMR”, reduct.marker.size”, and this process was repeated 100 times under fivefold cross-validation. The specific process was performed through the R package “BWGS” [39].

### 2.5. GS Practices with Different Population Sizes and Proportions

In this study, based on the size of the two populations, six different population sizes were set in the order of 604, 520/500, 400, 300, 200, 100, to compare the impact of population size on PA, five in the 60 K population and six in the WGR population, respectively. The RR-BLUP model was used for comparison; four-fifths of the population size was selected as the TP, and the BP was selected from the remaining samples of the total population, with one-fifth of the setting population size. This process was performed through the R package “rrBLUP” [20], which was repeated 100 times. At the same time, seven different proportions of TP and BP were tested, including 4:1, 3:1, 2:1, 1:1, 1:2, 1:3, and 1:4, which were also completed by the RR-BLUP model and repeated 100 times.

The rrBLUP model is described as:

y = Xβ + Zu + ε

where y is the vector of phenotypic traits, ε is the vector of random residuals with variance of Iσ^2^_ε_, β represents the fixed effects, and u represents the random effects with u~N (0, Kσ^2^_u_), where K is an identity matrix.

### 2.6. GS Practices That Include Nonadditive Effects

The additive, dominant, and epistatic effects of the markers were calculated using the R package “sommer” with reference to Li et al. [14]. The RKHS model was applied to the GS of additive, additive and dominance, and additive, dominance, and epistatic effects, which were implemented using the R package “BGLR” [40]. This was repeated 100 times.

### 2.7. GS Practices for Trait-Specific SNPs

The PCA analysis of genotypes was performed by PLINK [38]. Then, GWAS was performed using a mixed linear model with the first three principal components and kinship according to the phenotypic values and genotypes of the samples in TASSEL 5 [41]. SNPs were filtered according to *p*-values less than 0.1, 0.01, and 0.001, respectively. Markers with different threshold values were extracted by PLINK1.9 [38]. The GS practices for trait-specific SNPs were conducted using the R package “BWGS” with the GBLUP model, which was repeated 100 times.

### 2.8. GS Practices That Include Deleterious Mutations

The SIFT (sorting intolerant from tolerant) algorithm, by predicting whether an amino acid substitution is deleterious, bridges the gap between mutations and phenotypic variations. In this study, SIFT 4G software was applied to construct a database of deleterious mutations based on the *B. napus* reference genome ‘Darmor-bzh’ [2], and the SNPs of the 60 K and WGR populations were annotated [42]. SNPs with scores less than 0.05 were further identified as deleterious mutations. GS practices that include deleterious mutations in *B. napus* were implemented using the GBLUP model with 100 repetitions.

### 2.9. Statistical Analysis

Tukey’s tests in one-way ANOVAs were used to compare differences between methods, marker densities, population sizes, and proportions, as well as the inclusion of trait-specific SNPs and deleterious mutations; *p* < 0.05 was assumed to indicate a statistically significant difference. Comparisons between the nonadditive effects and additive effects were conducted by two-tailed unpaired Student’s *t* tests.

## 3. Results

### 3.1. PA of Ten Traits Under Four GS Models

**Figure 1:**
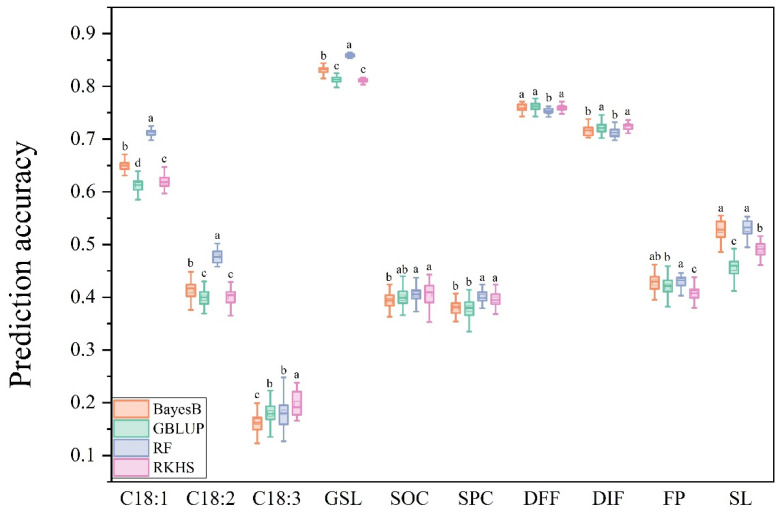
PA of the ten traits based on the four models. C18:1: oleic acid, C18:2: linoleic acid, C18:3: linolenic acid, GSL: glucosinolate, SOC: seed oil content, SPC: seed protein content, DFF: days to final flowering, DIF: days to initial flowering, FP: flowering period, SL: silique length. Different lowercase letters indicate the significant difference among different models at *p* < 0.05 by one-way ANOVA with Turkey’s test.

The PA achieved by four different models showed variation across ten traits. Among these traits, seven traits—C181, C182, GSL, SOC, SPC, FP, and S—achieved the best predictions when using the RF model (Figure 1). For the other three traits—C183, DFF, and DIF—the RKHS model provided the best predictions (Figure 1). Additionally, the RKHS model also presented optimal predictions for SOC and SPC (Figure 1). However, it performed poorly in predicting C181, C182, GSL, and FP. Both BayesB and GBLUP models achieved the best predictions for only two traits, which were DFF and SL for BayesB, DFF and DIF for GBLUP, respectively (Figure 1).

Among the ten traits, GSL had the highest PA, reaching 82.8%, followed by DFF and DIF, with accuracies of 75.8% and 71.9%, respectively. C18:3 had the lowest PA at only 18.1%. The PA for the remaining traits ranged from 38% to 65% (Table S3).

### 3.2. Effects of Different Marker Densities on PA

**Figure 2:**
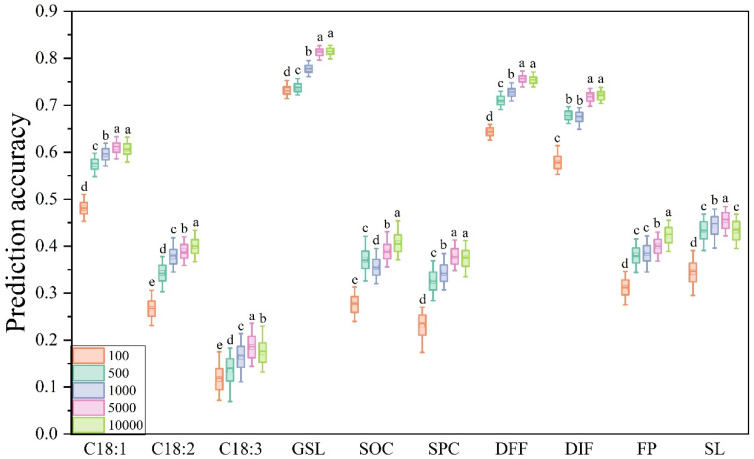
PA of ten traits based on different marker densities. Different lowercase letters indicate the significant difference among different marker densities at *p* < 0.05 by one-way ANOVA with Turkey’s test.

With increasing marker density, the PA tended to increase. Among the five marker densities, using 10,000 SNPs performed best prediction of eight traits, except for C18:3 and SL, while the use of 5000 SNPs for the prediction achieved the highest accuracy (Figure 2). In addition, for the prediction of C18:1, GSL, SPC, DFF, and DIF, 5000 SNPs achieved the same accuracy as 10,000 SNPs. There is no doubt that the PA decreased significantly when 100 SNPs were used, and as the number of markers was reduced from 5000 to 1000, the PA of all ten traits declined significantly. However, for 1000 and 500 SNPs, 500 SNPs performed relatively better when predicting SOC, while for DIF and FP, the results were comparable for both marker densities (Figure 2).

### 3.3. Effects of the Population Size and Ratio of TP to BP on the PA

**Figure 3:**
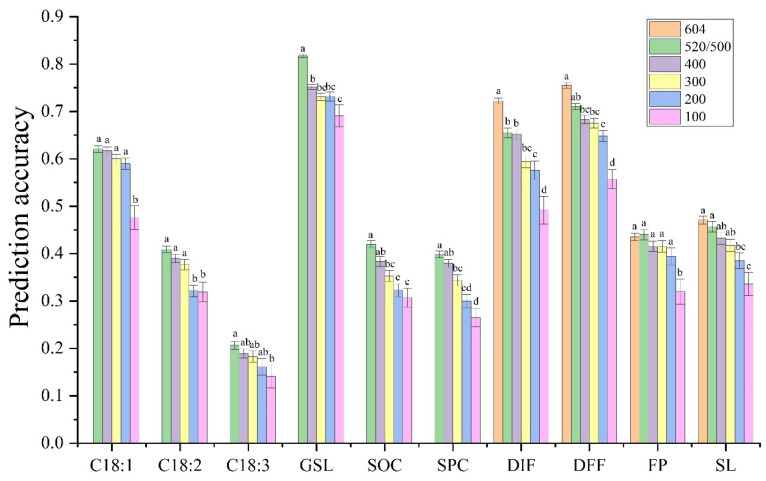
PA of ten traits based on different population sizes. The four traits (DIF, DFF, FP, and SL) corresponding to the WGR population were set to six population sizes, namely, 604, 500, 400, 300, 200, and 100. And, six traits corresponding to the 60 K population (C18:1, C18:2, C18:3, GSL, SOC, SPC) were set to five population sizes, namely, 520, 400, 300, 200, and 100. The values are the means ± SEs. Different lowercase letters indicate the significant difference among different population sizes at *p* < 0.05 by one-way ANOVA with Turkey’s test.

The different traits have different demands on population size to achieve maximum PA, and the ability to predict ten traits increased with increasing population size. However, only the PA of DIF and GSL were significantly different between the maximum population size set by this study and the next size (Figure 3). For C18:1, C18:3, and FP, the PA decreased significantly only when the population size was reduced to 100. When the population size decreased from 520/500 to 400, there was no significant difference in the PA of the nine traits, except for GSL. When the population size decreased to 300, the PAs of C18:1, C18:2, C18:3, FP, and SL exhibited no significant difference from the highest PA. And when the population size decreased from 300 to 200, the PA of C18:2 was significantly different.

**Figure 4:**
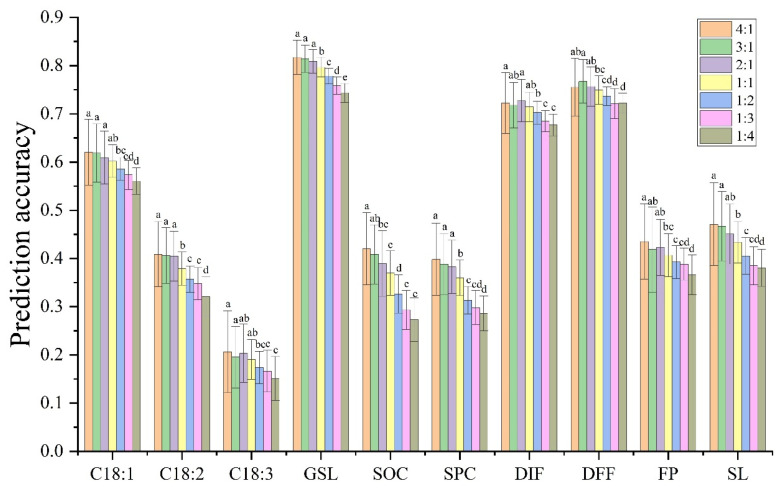
PA of ten traits based on different ratios of TP to BP. The values are the means ± SDs. Different lowercase letters indicate the significant difference among different ratios of TP to BP at *p* < 0.05 by one-way ANOVA with Turkey’s test.

The ratio of TP to BP also affected the PA. In this study, seven ratios were used: 4:1, 3:1, 2:1, 1:1, 1:2, 1:3, and 1:4. With the reduction in the proportion of TP, the PA tended to decrease. Notably, the PA of the ten traits did not differ significantly when the ratio of TP to BP was 4:1 or 3:1 (Figure 4). At the same time, the PA of nine traits, except SOC, did not decrease significantly when the ratio changed to 2:1. And the PA of three traits, C18:1, C18:3, and DIF, decreased significantly only when the ratio of TP to BP reached 1:2 (Figure 4).

### 3.4. The Influence of Nonadditive Effects on PA

**Figure 5:**
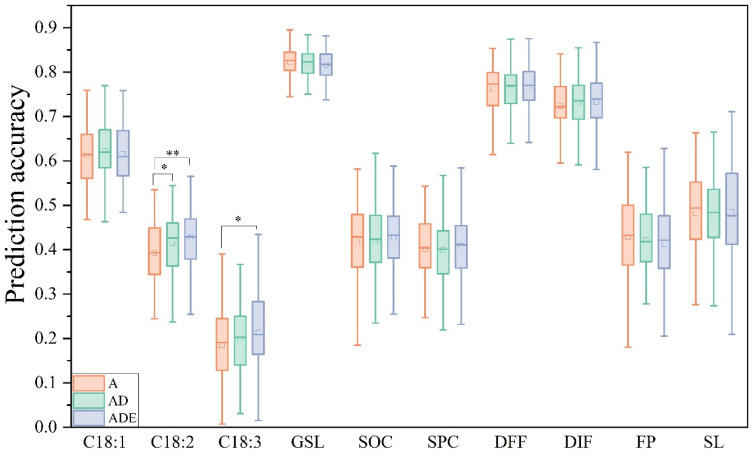
Effects of nonadditive effects on PA. A: additive, D: dominant, E: epistatic, AD: additive and dominant, ADE: additive, dominant, and epistatic. Differences in the PA of GS with and without nonadditive effects were analyzed by two-tailed Student’s *t* tests. *, *p* < 0.05, **, *p* < 0.01.

Previous studies have shown that nonadditive effects play an important role in the regulation of traits, especially in polyploids [29,30,31,32]. In this study, the RKHS model was used to compare the additive effects, additive and dominant effects, and additive, dominant, and epistatic effects on PA. The results showed that only C18:2 and C18:3 had significant differences in PA when nonadditive effects were added (Figure 5). The PA of C18:2 increased by 5.9% when the dominant effect was added and increased by 9.2% when the dominant and epistatic effects were added. After adding dominant and epistatic effects, the PA of C18:3 increased by 15.8%. Although there was no significant difference, the PA of C18:1 improved by 1.7% after the addition of the dominant effect. For SOC prediction, after adding dominant and epistatic effects, the PA increased by 1.9%. However, for FP and GSL, the PA decreased after the nonadditive effects were added.

### 3.5. Effect of Trait-Specific SNPs on PA

**Figure 6:**
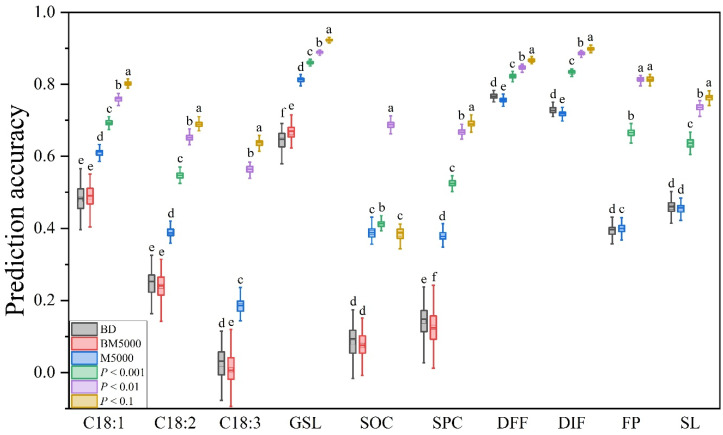
Effects of trait-specific SNPs and deleterious mutations on PA. BD: deleterious mutations in *B. napus*; BM5000: 5000 random markers in a population in which ten traits were measured; M5000: 5000 random markers in the 60 K and WGR populations. *p* < 0.1, *p* < 0.01, and *p* < 0.001 represent GS based on markers filtered by the *p*-values obtained by GWAS less than 0.1, 0.01, and 0.001, respectively. Different lowercase letters indicate the significant difference among different markers at *p* < 0.05 by one-way ANOVA with Turkey’s test.

In this study, markers with *p*-values less than 0.1, 0.01, and 0.001 according to GWAS were selected for GS. It can be seen that the predictive ability of nine traits (excluding SOC) was improved by filtering the markers according to the *p*-values. And, the PA achieved by the different markers also showed a significant difference, with maximum PA occurring when SNPs filtered by *p*-values less than 0.1 were used for GS. Among the nine traits, only FP yielded no significant difference in PA when using markers with *p*-values less than 0.1 and 0.01, and the PA of the other traits decreased with decreasing *p*-values (Figure 6). Since the numbers of markers obtained differed according to different *p*-values, to determine their ability to gain PA, we compared them with the adjacent marker density among the five marker densities set by the above analysis. The results showed that for traits with low PA, the gain effect was more obvious when SNPs filtered by *p*-values were applied for GS, such as C18:2, C18:3, FP, and SPC, and their average gain effects were greater than 90% (Table S4). In terms of the *p*-values, C18:1, C18:2, SPC, and DIF obtained the maximum gain effects at 0.001; C18:3, GSL, SOC, and FP had the greatest gains at 0.01; and DFF and SL had the highest gains at 0.1 (Table S4). We also analyzed the correlation between the number of markers obtained by different *p*-values and the PA (Table S5). The results showed that when filtered by *p*-values less than 0.01, the number of markers was positively correlated with the PA of traits, the correlation coefficient was 0.654 (*p* = 0.04) (Figure S1).

### 3.6. Effect of Deleterious Mutations on PA

Recent studies have shown that using deleterious mutations can improve the PA of GS. We used SIFT software to analyze the deleterious mutation from the WGR population and obtained the deleterious mutation sites (with a number of 5852), but few deleterious mutations were identified in the 60 K population. Therefore, six traits from the 60 K population corresponded to the WGR population, and 199 varieties with both deleterious mutation information and phenotypic data were obtained (Table S6). We compared the PA of the new population with 5000 randomly selected markers and deleterious mutations to that of the 60 K population with 5000 markers. Due to the decrease in population size, the PA for the six traits decreased in the 199 varieties, especially for C18:3 and SOC, for which the *p*-values were lower than 0.1 (Figure 6). Among the ten traits, compared with randomly selected SNPs, when using deleterious mutations for prediction, DFF, DIF, and SPC showed improvements of 1.4%, 1.4% and 13.4%, respectively. However, the PA of FP and GSL decreased by 1.4% and 3.2%, respectively.

## 4. Discussion

Different statistical models employ distinct approaches for GS. Among them, GBLUP directly estimates the GEBV using the genomic relationship matrix, which is stable and efficient [19]. RR-BLUP and BayesB, on the other hand, first estimate the effects of markers based on the TP and then compute the GEBV for individuals in the BP, with different assumptions on the variance of the marker effects [20,21]. RKHS and RF are semi-parametric and machine learning approaches, respectively [22]. This study compared the PA of BayesB, GBLUP, RF, and RKHS for ten traits and found that RF exhibited superior performance. In *B. napus*, among RR-BLUP, BayesCπ, EG-BLUP, and GBLUP models, RR-BLUP achieved the highest average PA for multiple seed quality traits [10], but the study did not compare semi-parametric and non-parametric models. In rice, Jacquin et al. [43] evaluated the predictive ability of different models for 15 traits and found that RKHS and the machine learning method SVR (Support Vector Regression) generally provided the most accurate predictions, aligning with this study. Furthermore, one study also highlighted that the RF model provided the highest PA for plant height and flowering time [12]. In this study, the RF model achieved the best prediction for 7 out of 10 traits, indicating that the performance of the RF model is not limited by the different characteristics of traits; hence, improving PA by selecting or optimizing the GS model is reliable.

In terms of predicting specific traits, according to the data in this study, GS showed similar prediction abilities for DIF and DFF, and close to the highest PA of the earlier GS study on flowering time [9]. The PAs of C18:1, C18:2, and C18:3 decreased successively in this study, which was consistent with the heritability of the three traits in different years and places presented in a previous study [11]. Generally, the PA followed a similar trend to genetic heritability. Traits with higher heritability have higher PA [13]. On the other hand, this study showed lower PA for SOC and SPC compared to previous research, but presented stronger PA for GSL [10]. This suggests that the type of population influences the PA of traits. This study selected natural populations as research subjects; the PA of the bi-parental DH population is more stable.

The comparison of PA with different marker densities has been a focus of many GS studies. In this study, five marker densities were used for comparison, which was consistent with the results of previous studies [24,26]. With the increasing marker density, the PA tended to increase. However, it is not the case that the greater the marker density was, the greater the PA. There was a limit value beyond which the PA no longer increased but even decreased. This phenomenon was observed for eight traits, for which the PA with 5000 markers was comparable to or greater than that with 10,000 markers. GS based on multiple rice traits also showed that when the number of markers reached a plateau, increasing the number of markers did not cause significant differences in PA [12]. A GS test on 575 rice hybrids also showed that the prediction ability reached a plateau at five K SNPs [44]. Thus, this study showed that 10,000 or 5000 markers can be used for effective GS application in *B. napus*. This also means that the cost of obtaining the required markers can be relatively lower, and the availability of GS in *B. napus* can be greater. However, due to the required time difference in different GS models, only the GBLUP model was used for comparison in this study, and more models can be used for subsequent analysis.

The PA of GS generally improved with increasing TP size because the larger the TP is, the more phenotypic and genotypic information can be used, which can correspondingly improve the accuracy of genetic effect estimation [45,46]. In this study, by comparing the PA of ten traits under different population sizes, it was found that it significantly differed between the largest and the second population size in two traits (DIF and GSL) with high PA. However, even though some traits exhibited large numerical changes, there was no significant difference between the two population sizes for the other eight traits. This finding showed that high PA also requires the support of a large population. A population of 400 samples seems to be the best size to choose, with a PA close to the largest population size or a decrease in only one difference level. With the increase in the proportion of BP, the PA showed a decreasing trend. However, the change in average PA caused by this strategy was smaller than that caused by changes in marker number and population size. Generally, when using a TP with a size three times the BP, the PA was the highest for all traits.

In this study, the application of nonadditive effects improved the prediction ability of C18:2 and C18:3, but the effect on other traits was weak. The reason for this weak improvement in PA may be the complex population diversity and the limited interpretability of nonadditive effects on the studied traits. Previous studies have shown that adding nonadditive effects to the GS model significantly improved the PA in *B. napus*, but in the case of wider population diversity, the ability to use dominant and epistatic effects decreased [47]. In a study of 109 maize inbred lines, the PA of most traits was not improved by the addition of nonadditive effects [14], but several other studies in maize also supported the view that combining nonadditive effects would improve the PA [48,49]. One possible reason for this was that the relationships between additive, dominant, and epistatic affinity matrices in the study of maize inbred lines were not independent, and the nonadditive variance can only provide limited new information; however, it can increase the noise of the prediction model, leading to a decrease in the predictive ability [14].

Compared with QTL mapping and GWAS, which play significant roles in exploring genetic mechanisms by identifying candidate genes for the target traits, the identified candidate genes can also be utilized for gene editing or molecular marker breeding. GS can not only capture the small effects of markers by optimizing models but also facilitate their application in breeding programs, especially in shortening the breeding cycle. In the GS of soybean oil content and protein, using markers filtered by the *p*-values according to GWAS was more accurate than random markers, and the PA was highest when markers were filtered by *p*-values less than 0.1 [50]. In maize, mixing trait-specific SNPs into GS models significantly improved the PA of southern leaf blight and gray leaf spot resistance [51]. In this study, the PA of all traits was improved by applying filtered markers based on *p*-values according to GWAS. The highest PA was achieved when markers were filtered with *p*-values less than 0.1, possibly due to the higher marker numbers. As the number of markers that could be obtained decreased with *p*-values less than 0.01 and 0.001, the greatest improvement in PA was observed when using markers filtered by *p*-values less than 0.01 and 0.001 compared to a similar number of random markers. Notably, through correlation analysis between the number of markers obtained with different *p*-values and the PA, our study identified that the number of markers filtered by *p*-values less than 0.01 was significantly and positively correlated with the PA. Therefore, our study provides a new index to evaluate the PA of one trait.

The presence of deleterious mutations led to decreased plant fitness, retarded growth, decreased fertility and yield [52]. Wu et al. [33] applied phylogenetic strategies to identify deleterious mutations in the potato genome and included deleterious mutations in GS, thus significantly improving the PA of yield and plant height. In this study, the PA of three traits (DFF, DIF, and SPC) was improved, but the PA of two traits (FP and GSL) decreased after deleterious mutations were included. The improvement and decrease in PA were minor. This may be related to the method used for deleterious mutation prediction. SIFT software was used in this study to characterize SNPs based on whether they affect protein function [42], and these SNPs may be evenly distributed in the genome without specificity for traits. Subsequent studies could further identify deleterious mutations in *B. napus* and assess their impact on GS based on phylogenetic analyses of Brassicaceae from broader genomic data.

In this study, the practice of GS was conducted using an associated population as the research object. While this approach has both advantages and disadvantages, it leverages the rich genetic diversity of the population, providing abundant genetic resources and minimizing artificial intervention. However, it also has a complex genetic structure, and compared to traditional breeding populations, achieving breeding goals would be relatively slow. In this study, we comprehensively evaluated the impact of various factors on GS based on the associated population in rapeseed. It broadens the application of GS and provides valuable insights for future implementation of GS in *Brassica napus*. It would also serve as a reference for optimizing breeding strategies in rapeseed improvement.

## 5. Conclusions

As an effective breeding strategy, GS has been widely used in plants. It is highly important to evaluate the influencing factors of the PA and determine the optimal combination of advantages, which can accelerate the practical application of GS. In this study, the PA of ten phenotypic traits in *B. napus* was compared under different GS models, marker densities, population sizes and proportions, and the application of nonadditive effects, trait-specific SNPs, and deleterious mutations. Finally, a model was established. First, the overall prediction ability of these traits could be evaluated based on the number of markers filtered by *p*-values obtained by GWAS less than 0.01, and a total population size of 400 samples or a TP three times greater than the BP should be further applied. Then, markers with *p*-values less than 0.1 could be used to carry out GS in the RF model to achieve the highest PA. Moreover, the application of nonadditive effects and deleterious mutations seemed unnecessary for traits with high PA but effective for traits with low PA.

## Data Availability

The relevant data supporting the conclusions of this study are included within the Supplementary Materials. And the raw datasets analyzed are available from the corresponding author on reasonable request.

## Supplementary Materials

The following supporting information can be downloaded at: https://www.mdpi.com/article/10.3390/plants14142095/s1. Supplemental Figure S1: Correlation between PA and the number of markers filtered by different *p*-values. MN0.001, MN0.01, and MN0.1 represent the number of markers filtered by *p*-values less than 0.001, 0.01, and 0.1, respectively. The numbers in the circles represent the Pearson correlation coefficient between the traits. Supplemental Table S1: Detailed information of the accessions in the 60 K population. Supplemental Table S2: The detailed information of the accessions in the WGR population. Supplemental Table S3 PA of ten traits based on four models. Supplemental Table S4: Gain effects of PA under different *p*-values. Supplemental Table S5: The number of markers under different *p*-values. Supplemental Table S6: Phenotypic values of 199 varieties.
