# Supplementary material for: Multifactor Analysis of a Genome-Wide Selection System in Brassica napus L
Source: Plants (Basel). 2025 Jul 8;14(14):2095. doi: 10.3390/plants14142095 (PMC12300503; doi:10.3390/plants14142095)
Supplement: Supplementary file 1 [file plants-14-02095-s001.zip › plants-3623413-supplementary figure S1.pdf]

plants-3623413-supplementary figure S1

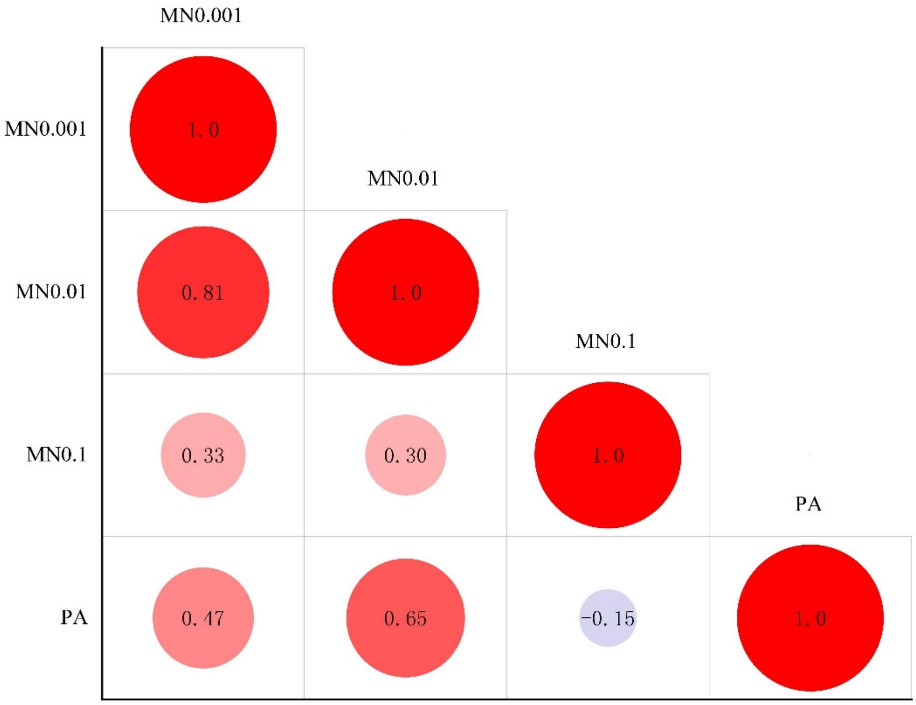

Fig. S1 Correlation between PA and the number of markers filtered by different *P* values. MN0.001, MN0.01, and MN0.1 represent the number of markers filtered by *P* values less than 0.001, 0.01, and 0.1, respectively. The numbers in the circles represent the Pearson correlation coefficient between the traits.
